# Female with Atraumatic Abdominal Bruising

**DOI:** 10.5811/cpcem.1247

**Published:** 2023-08-04

**Authors:** Zachary S. Pacheco, Grace Johnson, Erin F. Shufflebarger

**Affiliations:** University of Alabama Birmingham Heersink School of Medicine, Department of Emergency Medicine, Birmingham, Alabama

**Keywords:** Lupus erythematous panniculitis, dermatology

## Abstract

**Case presentation:**

We describe the case of a 38-year-old female patient with a history of lupus presenting with atraumatic abdominal pain and ecchymosis. The ultimate diagnosis of abdominal lupus erythematous panniculitis was determined based on physical exam and imaging findings.

**Discussion:**

Lupus erythematous panniculitis is a rare diagnosis, but consideration is important as early recognition and treatment is important to reduce pain and lessen the possibility of irreversible disfigurement and unnecessary costs to affected patients.

## CASE PRESENTATION

**Image 1:**
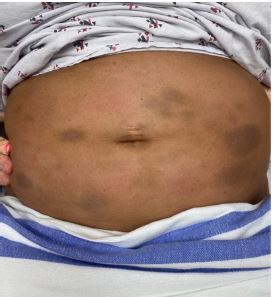
Scattered ecchymosis and nodularity seen on the patient’s abdominal physical exam.

**Image 2:**
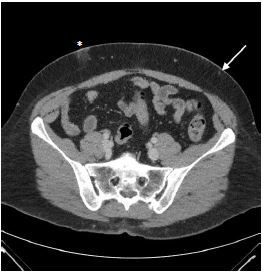
Computed tomography of the abdomen demonstrating areas of subcutaneous stranding and edema (*), as well as scattered areas of nodularity (arrow) in the soft tissues of the abdominal wall.

A 38-year-old female with a history of cutaneous lupus erythematosus on hydroxychloroquine presented to the emergency department (ED) with months of abdominal pain and two weeks of abdominal ecchymosis with underlying palpable nodules. She endorsed nausea without emesis, small-volume bowel movements, and shortness of breath secondary to pain. She denied similar previous episodes of this bruising or pain. However, the patient reported that her mother also had lupus with similar symptoms. Her abdominal exam was notable for generalized tenderness, diffusely scattered ecchymosis with palpable subcutaneous nodules (Image 1). Laboratory findings were unremarkable. Abdominal computed tomography was performed (Image 2).

The patient’s exam and imaging findings led to the diagnosis of lupus erythematous panniculitis (LEP) of the abdomen. After this diagnosis, she was discharged with a short course of steroids. At follow-up with her primary care physician weeks later, abdominal nodules were noted to be smaller in size and no longer tender.

## DISCUSSION

This case describes a rare ED diagnosis of abdominal LEP. This condition is a rare variant of systemic lupus erythematosus, occurring in approximately 2% of patients with this condition; however, it can also be seen in association with discoid lupus erythematosus or in isolation.^2^ Most lesions have been described as affecting the proximal extremities, face, and back of middle-aged females with a prior diagnosis of lupus.^2^ In patients with history and clinical exam suggestive of LEP, imaging including ultrasound, computed tomography, or magnetic resonance imaging can be used to evaluate for underlying abscess and for further characterization of the lesions.^3^ However, biopsy and histopathology are recommended for definitive diagnosis.^3^

The diagnosis of LEP is often delayed for greater than one year, which can lead to preventable complications such as calcification or atrophy. Treatment often requires two or more systemic therapies including first-line hydroxychloroquine; recurrence is common.^4^ It is important that emergency physicians be aware of this rare diagnosis and consider it when evaluating patients with relevant medical history, as timely diagnosis and treatment is important for pain reduction, irreversible disfigurement, and costs for affected patients.^4^

**CPC-EM Capsule**

What do we already know about this clinical entity?

*Lupus erythematous panniculitis (LEP) is rare, with lesions typically affecting the proximal extremities, face, and back of middle-aged females with a prior lupus diagnosis.*

What is the major impact of the image(s)?

*In patients with history and clinical exam suggestive of LEP, imaging aids evaluation for underlying abscess and further characterization of the lesions.*

How might this improve emergency medicine practice?

*Timely diagnosis and treatment is important for reducing pain and avoiding irreversible disfigurement and costs for affected patients.*
